# CCAAT/Enhancer-Binding Protein ε^27^ Antagonism of GATA-1 Transcriptional Activity in the Eosinophil Is Mediated by a Unique N-Terminal Repression Domain, Is Independent of Sumoylation and Does Not Require DNA Binding

**DOI:** 10.3390/ijms222312689

**Published:** 2021-11-24

**Authors:** Monika J. Stankiewicz, Jian Du, Dominick Martinico, Steven J. Ackerman

**Affiliations:** 1 Department of Biochemistry and Molecular Genetics, College of Medicine, University of Illinois at Chicago, Chicago, IL 60607, USA; monikastankiewicz2016@u.northwestern.edu (M.J.S.); jiandu82@gmail.com (J.D.); dmarti76@uic.edu (D.M.); 2 UW Carbone Cancer Center, University of Wisconsin School of Medicine and Public Health, Madison, WI 53705, USA; 3 College of Medicine, University of Illinois at Chicago, Chicago, IL 60607, USA

**Keywords:** eosinophil, granulocyte, transcription factor, repressor domain, sumoylation, terminal differentiation, HIV-tat, CCAAT/enhancer binding protein

## Abstract

CCAAT/enhancer binding protein epsilon (C/EBPε) is required for eosinophil differentiation, lineage-specific gene transcription, and expression of C/EBPε^32^ and shorter 27kD and 14kD isoforms is developmentally regulated during this process. We previously defined the 27kD isoform (C/EBPε^27^) as an antagonist of GATA-1 transactivation of the eosinophil’s major basic protein-1 (MBP1) P2-promoter, showing C/EBPε^27^ and GATA-1 physically interact. In the current study, we used a Tat-C/EBPε^27^ fusion protein for cell/nuclear transduction of an eosinophil myelocyte cell line to demonstrate that C/EBPε^27^ is a potent repressor of MBP1 transcription. We performed structure-function analyses of C/EBPε^27^ mapping its repressor domains, comparing it to C/EBPε^32^ and C/EBPε^14^, using GATA-1 co-transactivation of the MBP1-P2 promoter. Results show C/EBPε^27^ repression of GATA-1 is mediated by its unique 68aa N-terminus combined with previously identified RDI domain. This repressor activity does not require, but is enhanced by, DNA binding via the basic region of C/EBPε^27^ but independent of sumoylation of the RDI core “VKEEP” sumoylation site. These findings identify the N-terminus of C/EBPε^27^ as the minimum repressor domain required for antagonism of GATA-1 in the eosinophil. C/EBPε^27^ repression of GATA-1 occurs via a combination of both C/EBPε^27^-GATA-1 protein–protein interaction and C/EBPε^27^ binding to a C/EBP site in the MBP1 promoter. The C/EBPε^27^ isoform may serve to titrate and/or turn off eosinophil granule protein genes like MBP1 during eosinophil differentiation, as these genes are ultimately silenced in the mature cell. Understanding the functionality of C/EBPε^27^ in eosinophil development may prove promising in developing therapeutics that reduce eosinophil proliferation in allergic diseases.

## 1. Introduction

Human eosinophils and other granulocytes express up to four different isoforms from the C/EBPε gene, including 32, 30, 27 and 14kD isoforms [1] with potential activating and/or repressor functions based on the presence or absence of transactivation and putative repressor domains (Figure S1, Supplemental on-line data) [2]. The C/EBPε isoforms are generated both by alternative promoter usage (Pα versus Pβ) and RNA splicing, as well as alternative translational start sites [1,3]. The most well characterized isoform, full-length C/EBPε^32^, is approximately 94% similar to its murine counterpart [4]. Studies of C/EBPε deficient (knockout) mice which lack terminally differentiated eosinophils and neutrophils [5,6], and the identification of a homozygous recessive frameshift mutation in C/EBPε responsible for neutrophil specific granule deficiency [7,8], which also includes a deficiency in eosinophil development [9], demonstrate an essential role for the C/EBPε^32^ isoform in the terminal differentiation and maturation of granulocytes in both mice and humans, and the ability of this transcription factor to regulate various granulocyte promoters in a lineage-specific fashion [5,7,10,11]. We previously demonstrated that enforced expression of the full-length isoform, C/EBPε^32^, defaults hematopoietic progenitors to the eosinophil lineage at the expense of all other myeloid lineages regardless of the presence or absence of eosinophil-specific IL-5 or neutrophil/erythroid specific cytokines, while the shorter isoforms, C/EBPε^27^ and C/EBPε^14^, inhibit eosinophil differentiation and gene expression [12].

We previously reported that the ability of the C/EBPε^27^ isoform to inhibit eosinophil differentiation and gene expression occurs via its unique ability to repress GATA-1-mediated transactivation of eosinophil gene promoters, while the other activator and repressor isoforms, C/EBPε^32^ and C/EBPε^14^, lack this activity [11]. In addition, we showed that the C/EBPε^27^ isoform physically interacts with GATA-1 in vivo in studies using co-immunoprecipitation from nuclear extracts of an eosinophil myelocyte cell line using antibodies to either GATA-1 or C/EBPε [11]. GATA-1 has been shown to be critical for the development and differentiation of multiple myeloid lineages including erythroid, megakaryocyte, mast cell, and eosinophil [13,14]. Retroviral transduction of human CD34+ hematopoietic progenitors with a GATA-1 expression vector selectively drives these myeloid progenitors to differentiate nearly exclusively into eosinophils [13], and transgenic deletion of a palindromic double GATA-1 binding site in the murine GATA-1 HS2 control locus, leads to an exclusive loss of the eosinophil lineage [14], providing a novel eosinophil-deficient mouse for studies of eosinophil effector function in the pathophysiology of asthma [15] and other eosinophil-associated diseases. We previously reported that GATA-1, but not GATA-2, strongly activates the promoters of eosinophil-specific genes such as MBP1 [16] through synergy with C/EBPβ [17], leading to the hypothesis and subsequent studies in the avian system [18] that the combinatorial regulation of eosinophil-specific gene transcription during eosinophil differentiation requires the coordinate activities of transcription factors that include GATA-1 and PU.1 [11], and various members of the C/EBP family, particularly C/EBPε [5].

To further delineate the mechanism(s) by which the C/EBPε^27^ isoform antagonizes GATA-1 activity, we conducted a structure-function analysis to determine which domains in the C/EBPε^27^ isoform contribute to its repressor activity in comparison to the full-length C/EBPε^32^ activator, and shorter C/EBPε^14^ repressor isoforms. Transcriptional repression mediated by the RDI domain of the murine equivalent of full-length C/EBPε^32^ was reported to be dependent upon sumoylation of a conserved SUMO consensus site (VKEEP) [19]. The human C/EBPε^27^ isoform shares a segment of the C/EBPε^32^ RDI core domain [2] including this VKEEP sumoylation site. The murine 34kD ortholog of C/EBPε^32^ containing this SUMO consensus site is sumoylated in vivo [19], and this site is conserved among the various C/EBPε proteins identified thus far from human, rat, and sheep. Other C/EBP family members including C/EBPα and C/EBPβ also contain a SUMO consensus site, a common theme among the C/EBPs [19,20,21,22]. Thus, we have also addressed whether sumoylation plays a role in C/EBPε^27^ repression of GATA-1 transcriptional activity. Our results show that C/EBPε^27^ functions in vivo as a repressor of endogenous eosinophil gene transcription, that two repression domains contribute to its attenuation of GATA-1 activity—particularly its unique N-terminal domain, that repression of GATA-1 is independent of sumoylation of C/EBPε^27^, and that it does not require DNA binding to a proximal C/EBP site.

## 2. Results

### 2.1. C/EBPε and GATA-1 Bind In Vivo in Eosinophil Myelocytes to Their Functional Sites in the MBP1-P2 Promoter

**Figure 1:**
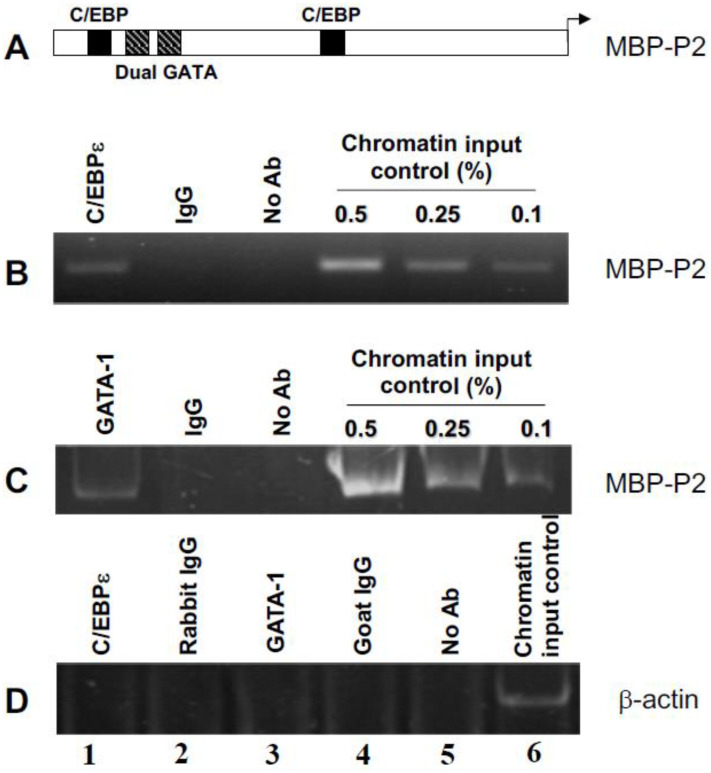
Chromatin immunoprecipitation of the MBP promoter from AML14.3D10 eosinophils demonstrates in vivo occupancy of the P2 promoter by C/EBPε and GATA-1. Structure of the MBP1-P2 promoter with two functional C/EBP sites and the high-affinity double GATA site is shown in (**A**). The MBP1-P2 promoter was analyzed by ChIPs (**B**,**C**) from nuclei of the AML14.3D10 eosinophil myelocyte line that constitutively expresses MBP1 mRNA and protein. ChIP analyses using antibodies to C/EBPε (**B**), and GATA-1 (**C**) demonstrate the in vivo binding of these factors to the endogenous MBP1-P2 promoter. Negative controls included non-immune IgG and no antibody addition as indicated. Amplification of the human β-actin gene was used as a negative control for the non-specific immunoprecipitation of DNA (**D**). Comparisons to chromatin input of 0.1–0.5% are indicated for the C/EBPε and GATA-1 ChIPs (**B**,**C**) and 0.5% for the β-actin control (**D**).

To demonstrate in vivo occupancy of the eosinophil MBP1-P2 promoter by both C/EBPε and GATA-1, we performed chromatin immunoprecipitation (ChIP) assays in the AML14.3D10 eosinophil myelocyte cell line to assess binding to their target sequences in the MBP1 gene. This human eosinophil cell line constitutively expresses all of the secondary granule proteins, including MBP1, present in terminally differentiated blood eosinophils, and it forms secondary granules morphologically equivalent to authentic blood eosinophils [23,24]. As shown in Figure 1, the ChIP analyses demonstrated in-vivo occupancy of the MBP1-P2 promoter by both C/EBPε and GATA-1 as compared to negative controls for the IP including non-immune IgG and no antibody, and PCR amplification of an unrelated gene, β-actin, to control for the non-specific IP of genomic DNA by the anti-GATA-1 or anti-C/EBPε antibodies.

### 2.2. Transduction with a TAT-C/EBPε^27^ Fusion Protein Inhibits GATA-1 Transactivation of the MBP1-P2 Promoter in CV-1 Cells and Expression of the Endogenous MBP1 Gene in AML14.3D10 Eosinophils

**Figure 2:**
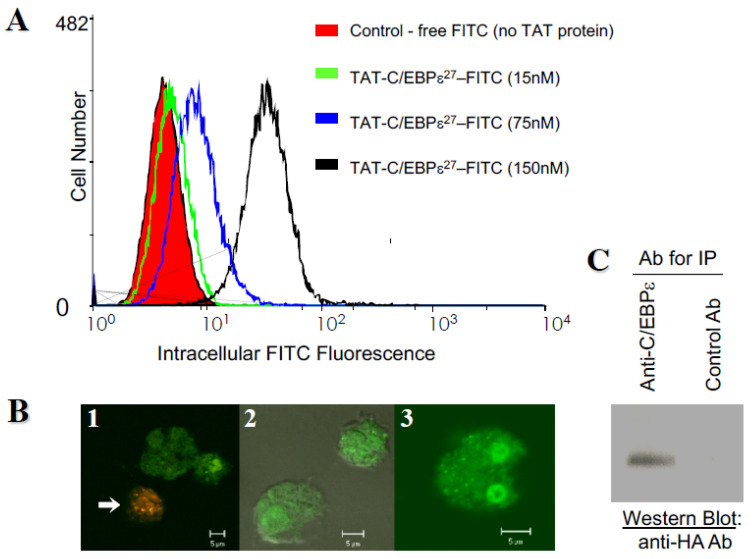
Transduction of the AML14.3D10 eosinophil myelocyte cell line by TAT-C/EBPε^27^. Flow cytometry of intracellular FITC fluorescence in AML14.3D10 eosinophils transduced with increasing doses of FITC-TAT-C/EBPε^27^ for one hr. (**A**). The pH of the cell suspension buffer and sheath fluid for the flow cytometer was adjusted to 6.8 to quench extracellular FITC fluorescence as previously described (26). Aliquots of the same cells used for flow cytometry were cytocentrifuged onto slides and analyzed by confocal microscopy for intracellular FITC fluorescence (**B**); images are shown for the 75 nM (panel 1) and 150 nM (panels 2–3) doses. A non-transduced cell in panel B1 shows autofluorescence characteristic of AML14.3D10 eosinophils (arrow). Both nuclear and cytosolic localization of the FITC-TAT-C/EBPε^27^ fusion protein is shown (panels B2 and B3). In (**C**), AML14.3D10 eosinophils were transduced with 1 μM TAT-C/EBPε^27^, lysed in immunoprecipitation buffer, and the cell lysate divided equally among samples that were either immunoprecipitated with antibodies to HA (the TAT-C/EBPε^27^ construct is tagged at its N-terminus with an HA epitope) or C/EBPε, control non-immune antibody or saved as the input control. Western blotting of the immunoprecipitates used an anti-HA antibody for detection of the fusion protein.

We previously reported that C/EBPε^27^ is a potent repressor of GATA-1 transactivation of the MBP1-P2 promoter in reporter gene assays in heterologous cell lines [11]. To demonstrate the ability of C/EBPε^27^ to repress endogenous MBP1 gene transcription in vivo in authentic eosinophil progenitors, we utilized HIV TAT-C/EBPε fusion proteins for high efficiency transduction of the AML14.3D10 eosinophil myelocyte cell line and studied their effects on transcription of the MBP1 gene. To first demonstrate that the TAT-C/EBPε^27^ fusion protein efficiently transduces the AML14.3D10 eosinophil cell line, the fusion protein was FITC-labeled, and the transduced cells analyzed by flow cytometry, confocal microscopy and immunoprecipitation with an anti-C/EBPε antibody (Figure 2). As shown by flow cytometry (Figure 2A), the FITC-conjugated TAT-C/EBPε^27^ fusion protein transduced AML14.3D10 cells in a dose-response fashion, with >99% of the cells being positive at 150 nM FITC-TAT-C/EBPε^27^ compared to a free FITC only (no TAT fusion protein) control. By confocal microscopy, both cytoplasmic and nuclear localization of the FITC-TAT-C/EBPε^27^ was evident at the higher concentrations of 75 and 150 nM used for the transductions (Figure 2B). To further demonstrate cellular uptake of the TAT-c/EBPε^27^ fusion protein by the AML14.3D10 eosinophil myelocytes, we transduced the cells and performed IP with an anti-C/EBPε antibody, followed by SDS-PAGE and Western blotting with an anti-HA tag antibody for detection of the transduced but not endogenous C/EBPε (Figure 2C). The anti-C/EBPε antibody, but not the non-immune control antibody, successfully immunoprecipitated the appropriate size fusion protein band as detected by the anti-HA antibody, further demonstrating successful transduction of the AML14.3D10 eosinophil cell line. In order to determine whether the transduced TAT-C/EBPε^27^ was functionally active in vivo and capable of antagonizing GATA-1 transactivation of the MBP1-P2 promoter, we first transduced CV-1 cells with TAT-C/EBPε^27^ or a TAT-GFP control protein, followed by transfection with the GATA-1 expression vector and MBP1-P2 promoter luciferase reporter, and compared these to non-transduced CV-1 cells transfected with GATA-1, the C/EBPε^27^ expression vector and MBP1-P2 promoter luciferase reporter plasmid (Figure S2, Supplemental on-line data). The TAT-GFP fusion protein used in these experiments as the negative control has previously been shown to efficiently transduce > 99% of purified blood eosinophils (26). Transduction of CV-1 cells with TAT-C/EBPε^27^ was highly effective in repressing GATA-1 transactivation of the MBP1-P2 promoter in a dose-response fashion (Figure S2A) to a maximum of ~50% (Figure S2B) as compared to the TAT-GFP fusion protein control at the highest concentration tested, with repressor activity for GATA-1 comparable to the C/EBPε^27^ expression vector in non-transduced cells (Figure S2A).

**Figure 3:**
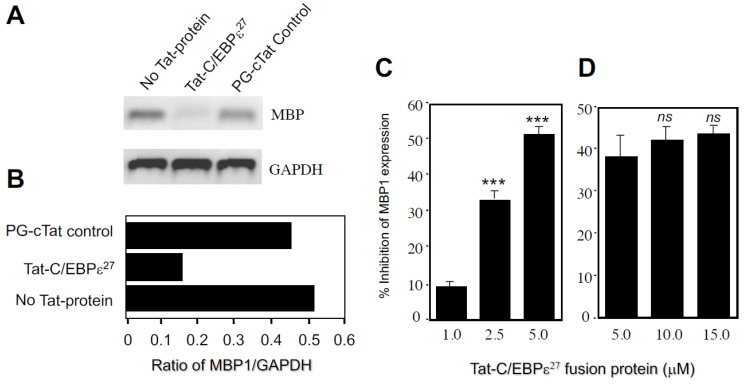
Transduction of AML14.3D10 eosinophils with a TAT-C/EBPε^27^ fusion protein induces dose-response inhibition of endogenous MBP1 gene expression. (**A**,**B**) AML14.3D10 eosinophil myelocytes (1 × 10^6^ cells) were transduced with 80 μg purified TAT-C/EBPε^27^ fusion protein (3.5 μM), a PG-cTAT fusion protein control (8.6 μM) or not transduced. Cells were harvested at 18 h, total RNA prepared, and MBP1 mRNA expression determined by semi-quantitative RT-PCR in a pre-standardized linear amplification range using GAPDH as control for equalization of cDNA inputs (**A**). Results analyzed by ethidium bromide staining were quantitated using ImageQuant™software and are plotted as the ratio of MBP1 to GAPDH signal intensity (**B**). (**C**,**D**) AML14.3D10 eosinophil myelocytes were transduced with purified TAT-C/EBPε^27^ fusion protein or a TAT-GFP fusion protein control. Cells were harvested at 8 h post transduction, total RNA prepared, and analyzed by Taqman™ Real Time RT-PCR assessment of steady state levels of MBP1 mRNA. Results are plotted as the % inhibition of MBP1 mRNA expression by TAT-C/EBPε^27^ relative to the TAT-GFP control transduced cells. TAT-C/EBPε^27^ inhibited endogenous MBP1 mRNA expression in a dose-response manner to an inhibition maximum of ~50% at 5 μM (**C**). Transduction with larger doses of TAT-C/EBPε^27^ (5–15 μM) did not further increase the inhibition of MBP1 gene expression beyond ~50% (**D**). Results (mean ± SD) in C and D are from two independent experiments. *** *p* < 0.001 compared to lowest concentration tested. *ns*, not significant.

**Figure 4:**
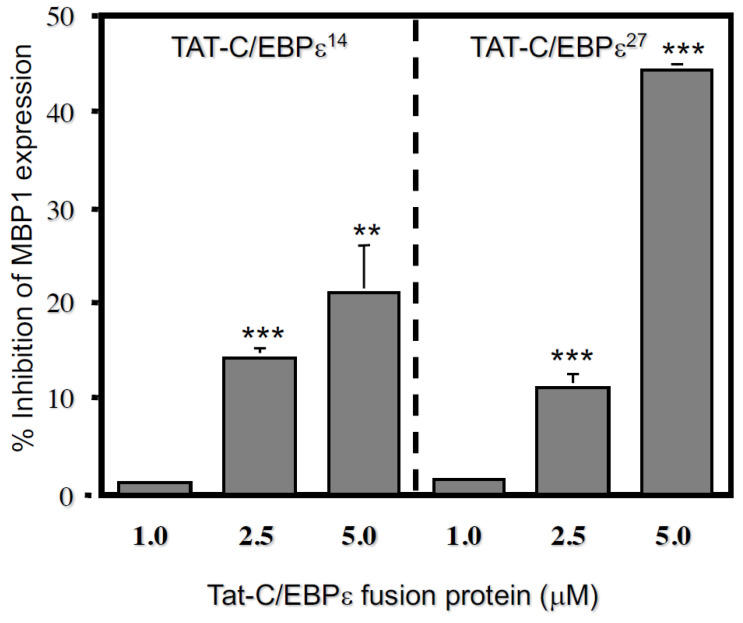
TAT-C/EBPε^27^ and TAT-C/EBPε^14^ inhibit endogenous MBP1 gene expression when transduced into AML14.3D10 eosinophil myelocytes. AML14.3D10 eosinophil myelocytes were transduced for 24 h with TAT-C/EBPε^14^, TAT-C/EBPε^27^, and TAT-GFP as control at equimolar concentrations. Two doses of the TAT-fusion proteins were given to cumulative final concentrations of 1.0, 2.5 and 5 μM over 24 h. Cells were harvested at 24 h, total RNA extracted, and used for quantitative Taqman™ Real Time RT-PCR assessment of steady state levels of MBP1 mRNA. Samples were analyzed for both MBP1 and GAPDH genes and compared to standard curves for each mRNA prepared from non-transduced AML14.3D10 cells analyzed under the same conditions except for the addition of the TAT-fusion proteins. The ratio of MBP1/GAPDH was determined for each sample, normalized to the value obtained for the TAT-GFP control transduced cells, and the results plotted as the mean (±SD) percent (%) inhibition of MBP1 mRNA expression compared to the TAT-GFP transduced control cells used for normalization of non-specific effects at the identical doses used for TAT-C/EBPε^27^ and TAT-C/EBPε^14^. In comparison to the TAT-GFP control, TAT-C/EBPε^27^ and TAT-C/EBPε^14^ inhibited MBP1 mRNA transcription by ~45% and ~22%, respectively. Representative results from 2 independent experiments are shown. ** *p* < 0.01, *** *p* < 0.001 compared to 1 μM.

We next tested whether the TAT-C/EBPε^27^ fusion protein could inhibit endogenous MBP1 gene expression. AML14.3D10 eosinophil myelocytes were transduced for 18 h. with TAT-C/EBPε^27^ or a control TAT-fusion protein (PG-cTAT) of similar size [25] and analyzed for effects on the steady-state levels of MBP1 mRNA using semi-quantitative RT-PCR (Figure 3A). The TAT-C/EBPε^27^ fusion protein transduced >99% of the AML14.3D10 eosinophils within ~60 min (data not shown) and significantly decreased steady state MBP mRNA levels by ~70% compared to the PG-cTAT and no TAT fusion protein controls at 18 h post transduction (Figure 3B). This experiment was repeated comparing dose-response inhibition of MBP1 mRNA expression by TAT-C/EBPε^27^ to a TAT-GFP control as measured by quantitative Real-Time RT-PCR (Figure 3C,D). The TAT-C/EBPε^27^ fusion protein significantly reduced MBP1 mRNA levels by a maximum of ~50% when tested for 8 h at 5 μM (Figure 3C), and higher doses up to 15 μM did not further inhibit MBP gene expression beyond ~50%. For comparison to TAT-C/EBPε^27^, we tested a TAT-C/EBPε^14^ fusion construct, since C/EBPε^14^ lacks a transactivation domain, should be competent to homodimerize and heterodimerize with the other C/EBPε isoforms or other C/EBP family members and bind to DNA, and is therefore hypothesized to function as a dominant negative inhibitor of C/EBP-mediated gene transcription [10,11,26]. As we previously reported, the C/EBPε^14^ isoform does not inhibit GATA-1 transactivation of the MBP1-P2 promoter but does inhibit both C/EBPα− and C/EBPβ−mediated transactivation [11] and would therefore be predicted to inhibit endogenous MBP1 gene expression. For these experiments, AML14.3D10 eosinophil myelocytes were transduced for 8 h with either TAT-C/EBPε^14^, TAT-C/EBPε^27^ or the TAT-GFP control protein and effects on MBP1 mRNA expression levels determined by quantitative Real-Time RT-PCR as above (Figure 4). As before, the TAT-C/EBPε^27^ fusion protein significantly reduced MBP1 mRNA levels by up to ~50% in a dose-response fashion. In contrast, C/EBPε^14^ was less active but still inhibitory, reducing MBP1 expression in a dose-response fashion by up to ~25% compared to the TAT-GFP control. These results support a negative regulatory role for these C/EBPε isoforms in eosinophil gene expression and demonstrate the utility of using TAT-transcription factor fusion proteins for analyzing transcription factor activities and mechanisms regulating myeloid gene expression in vivo.

### 2.3. Repression Domains of C/EBPε^27^ Attenuate GATA-1 Transcriptional Activity: Both the Unique C/EBPε^27^ N-Terminus (RD27) and RDI Domains Inhibit GATA-1 Activity

**Figure 5:**
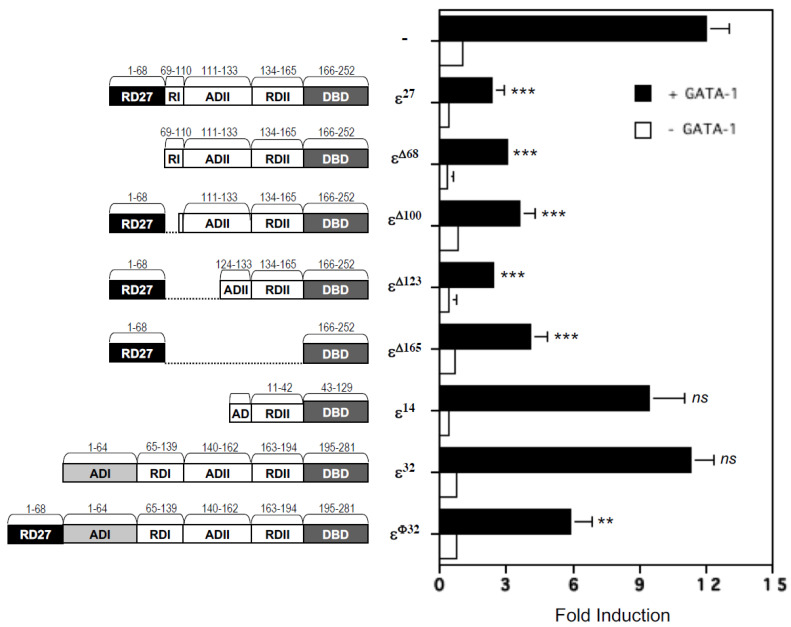
Mutational analyses of C/EBPε^27^ demonstrate that both its unique 68 amino acid N-terminal (RD27) and RDI domains are required for repression of GATA-1 activity for the MBP1-P2 promoter. The cartoons to the left show the unique N-terminal (RD27) and previously mapped repressor (RDI, RDII) and activation (ADI, ADII) domains shared amongst the various C/EBPε isoforms. Regions removed in the various deletion mutants and added in the fusion proteins are indicated. The DNA-binding domain (DBD) includes both the basic region (BR) and leucine zipper dimerization domain. Both the previously described RDI repression domain of C/EBPε^32^ (amino acids 64–129) for which its core sequence (amino acids 68–100) is conserved in the C/EBPε^27^ isoform, and the RD27 domain (distinct N-terminal 68 amino acids of C/EBPε^27^) contribute to C/EBPε^27^ antagonism of GATA-1 activity in transactivation assays of the MBP P2 promoter performed in CV-1 cells in the presence or absence of co-transfection with the GATA-1 expression vector. Both the Δ2–68 and Δ69–165 deletion mutants of C/EBPε^27^ retain their full inhibitory activity in co-transfections with GATA-1 compared to full length C/EBPε^32^, C/EBPε^27^, and C/EBPε^14^. The Δ69–100, Δ69–123, and Δ69–165 deletion mutants of C/EBPε^27^ retain their repressor activity even though the core/complete RDI domain of wild type C/EBPε^27^, including the sumoylation (SUMO) consensus site (amino acids 91–95 in C/EBPε^27^) is absent. Fusion of the unique RD27 domain to full length C/EBPε^32^ (ε^φ32^) converts it into a partial repressor of GATA-1. *** *p* < 0.001; ** *p*< 0.01; *ns*, not significant, compared to GATA-1 alone.

To map the repressor domain(s) in the C/EBPε^27^ isoform responsible for its potent antagonism of GATA-1 transcriptional activity, we generated a series of deletion and fusion mutation constructs in the C/EBPε^27^ expression vector. Figure 5 illustrates the conserved functional domains for the wild type isoforms of C/EBPε^32^, C/EBPε^27^, C/EBPε^14^ and the mutants of C/EBPε^32^ and C/EBPε^27^ generated for this purpose. Design of the mutant C/EBPε proteins was based on previously published work on the murine C/EBPε^32^ ortholog (20,32) and the human C/EBPε isoforms [1,2]. Results for co-transactivation assays of the MBP1-P2 promoter with GATA-1 combined with the wild type C/EBPε^32^, C/EBPε^27^, C/EBPε^14^ expression vectors, and the deletion mutants of the C/EBPε^27^ isoform, are shown in Figure 5. These analyses include a fusion protein of C/EBPε^32^ (C/EBPε^φ32^) in which the unique N-terminal 68 amino acids of C/EBPε^27^ are fused to the N-terminus of C/EBPε^32^. In the absence of GATA-1, none of the three wild type isoforms of C/EBPε or deletion mutants of C/EBPε^27^ showed any significant transactivating or inhibitory potential for the MBP1-P2 promoter, confirming our prior report [11]. In contrast, wild type C/EBPε^27^ potently inhibited GATA-1 transactivation of this promoter, whereas the C/EBPε^14^ isoform, nature’s own deletion of the unique N-terminus RD27, RDI, and most of the ADII domains of C/EBPε^27^, did not antagonize the activity of GATA-1. Deletion of the unique N-terminal RD27 domain alone from C/EBPε^27^, a mutant containing part of the RDI and the entire RDII domain was still capable of fully antagonizing GATA-1, suggesting participation of the RDI domain, since its absence in the C/EBPε^14^ isoform abrogates the inhibition of GATA-1 activity. Internal deletions of sequences between the N-terminal RD27 and b-ZIP/basic DNA binding domain of C/EBPε^27^ (ε^Δ69–100^, ε^Δ69–123^) retained their ability to antagonize GATA-1, including importantly the construct containing only the unique RD27 and b-ZIP/DNA-binding domains (ε^Δ69–165^), demonstrating repressor activity for this unique N-terminal 68 amino acid sequence not present in the other C/EBPε isoforms. Of interest, fusion of the RD27 domain to the N-terminus of full-length C/EBPε^32^ converted it into a repressor of GATA-1, further supporting its role in the suppressor activity of C/EBPε^27^. Based on these results, one of the minimum repression domains necessary for antagonism of GATA-1 includes the previously identified RDI domain of C/EBPε^32^, part of which is conserved and present in the C/EBPε^27^ isoform (Figure 5). However, removal of this “core” RDI domain, thought to be responsible for the full domain’s repressor activity (32), failed to diminish C/EBPε^27^ repressor activity as shown by the ε^Δ69–100^ mutant. Further internal deletions, such as ε^Δ69–123^, which results in the unique N-terminal 68 amino acid sequence of C/EBPε^27^ (RD27) being fused to the region of C/EBPε^27^ identical to the short C/EBPε^14^ isoform, and ε^Δ69–165^, which removes all of the intervening sequence between these N-terminal 68 amino acids of C/EBPε^27^ and its DNA binding domain (basic region responsible for DNA binding and the leucine zipper dimerization domain), both retained full repression of GATA transactivation of the MBP1-P2 promoter. Of note, the ε^Δ69–123^ and ε^Δ69–165^ mutants do not possess any of the RDI domain present in wild type C/EBPε^27^, nor do they possess the SUMO consensus site (VKEEP) present in both wild type C/EBPε^32^ and C/EBPε^27^. However, they do retain the unique N-terminal 68 amino acid region we have termed RD27. As this region is unique to the C/EBPε^27^ isoform and is not present in any of the other C/EBPε isoforms nor in any other C/EBP family members, it may act as the minimum domain necessary for C/EBPε^27^ inhibition of GATA-1 function in eosinophil progenitors.

### 2.4. C/EBPε Is Constitutively Sumoylated in Eosinophilic Myelocytes and in Heterologous Cells Transfected with a SUMO-1 Expression Vector

**Figure 6:**
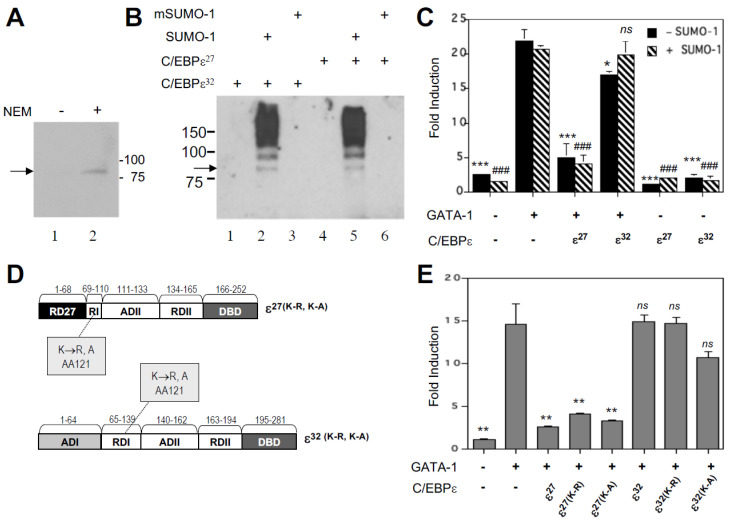
(**A**,**B**) C/EBPε is sumoylated in the eosinophil lineage. Detection of endogenous sumoylated C/EBPε in AML14.3D10 eosinophil lysates prepared in the presence of 50 μM *N*-ethylmaleimide, immunoprecipitated with anti-C/EBPε antibody, and detected by Western blotting using an anti-SUMO-1 antibody (**A**). Detection of sumoylated C/EBPε^32^ and C/EBPε^27^ in lysates of COS-7 cells transfected as indicated with expression vectors for C/EBPε^27^, C/EBPε^32^, FLAG-SUMO-1, and mutant-FLAG-SUMO-1 (a form of SUMO-1 in which the target glycine has been converted to alanine and is unavailable for conjugation) (**B**). Lysates prepared with 50 μM *N*-ethylmaleimide were immunoprecipitated with anti-C/EBPε antibody and the Western blot probed with anti-FLAG M2 HRP-conjugated antibody. (**C**–**E**) Over-expression of SUMO-1 or mutation of the C/EBPε^27^ sumoylation site has no effect on the repressor activity of C/EBPε^27^ for GATA-1 transactivation of the MBP1-P2 promoter. Co-transfection of a SUMO-1 expression vector with C/EBPε^32^ or C/EBPε^27^, does not alter the repressor activity of C/EBPε^27^ for GATA-1 trans-activation of the MBP1-P2 promoter, nor convert C/EBPε^32^ into a repressor independently or for GATA-1 (**C**) (* *p* < 0.05, ***, ### *p* < 0.001, compared to GATA-1 alone, ±SUMO-1, respectively). Mutation of the sumoylation target lysine (K) residue in the conserved SUMO (VKEEP) consensus site to either arginine (K→R) or alanine (K→A) (**D**) does not alter the repressor activity of C/EBPε^27^ for GATA-1 transactivation of the MBP1-P2 promoter (**E**) (** *p* < 0.01; *ns*, not significant, compared to GATA-1 alone). Mutation of this target lysine in full length C/EBPε^32^ (**D**) does not convert it into an activator or repressor in the presence of GATA-1 (**E**).

The human C/EBPε^27^ isoform contains the conserved “VKEEP” sequence first identified within the RDI domain of murine C/EBPε [27], which was subsequently shown to be a target for sumoylation as a requirement for its inhibitory activity [19]. To determine whether the human C/EBPε^27^ repressor isoform is similarly sumoylated, we used immunoprecipitation of endogenous C/EBPε from the AML14.3D10 eosinophil myelocyte cell line that expresses all of the human C/EBPε isoforms [11], followed by Western blotting with an anti-SUMO-1 antibody (Figure 6A). These analyses were performed in the presence or absence of the isopeptidase inhibitor *N*-ethylmaleimide (NEM), which has been shown to prevent cleavage of the SUMO-1 moiety upon cell lysis [28]. As shown in Figure 6A, in the presence but not absence of 50 mM NEM, we detected a single high molecular weight sumoylated form of C/EBPε. It is currently unclear which of the C/EBPε isoforms this represents, since isoform-specific antibodies are not commercially available and have been difficult to generate (Ackerman, unpublished results). The high molecular weight of the sumoylated C/EBPε may be due to migration of the modified form slower than expected for its size as has been seen for other sumoylated proteins including C/EBPα, C/EBPβ, and murine C/EBPε [19,20,21]. Though similar in size to that detected for murine C/EBPε, it is possible that human C/EBPε is polysumoylated, accounting for its slightly larger size. We performed a similar immunoprecipitation analysis in a C/EBPε negative heterologous cell line, COS-7, co-transfected with expression vectors for FLAG-tagged SUMO-1 and C/EBPε^32^ or C/EBPε^27^. Immunoprecipitation of the transfected COS-7 cell lysates with an anti-C/EBPε antibody, followed by Western blotting with an anti-FLAG-HRP conjugated antibody (M2) detected a similar size protein band as seen in AML14.3D10 eosinophil lysates (Figure 6B), as well as multiple higher molecular weight FLAG-tagged sumoylated species. The larger, slower migrating bands may represent additional poly-sumoylated forms of C/EBPε^32^ or C/EBPε^27^ due to the over-expression of SUMO-1 from the expression vector in transfected COS-7 cells, or alternatively the co-immunoprecipitation of other FLAG-tagged sumoylated proteins that interact with the C/EBPε isoforms.

### 2.5. Over-Expression of SUMO-1 Has No Effect on C/EBPε^27^ Inhibition of GATA-1 Transactivation of the MBP1-P2 Promoter

Studies by Williams and colleagues [19] suggested that the transcriptional repression mediated by the RDI domain of murine C/EBPε is mediated by the addition of SUMO-1 to the lysine in its core “VKEEP” SUMO consensus site. This SUMO consensus site is fully conserved in the RDI domain of human C/EBPε^27^. To determine whether sumoylation is also necessary for C/EBPε^27^ repression of GATA-1 activity for the MBP1-P2 promoter, a SUMO-1 expression vector was co-transfected into CV-1 cells along with the vectors for C/EBPε^32^, C/EBPε^27^, and GATA-1 (Figure 6C). The expression of SUMO-1 did not affect the repressor activity of C/EBPε^27^ for GATA-1, nor did it convert the C/EBPε^32^ isoform into an antagonist of GATA-1. The converse experiment was also performed to further address a possible role for sumoylation of C/EBPε^27^. Point mutations of the sumoylation target lysine residue in the VKEEP sequences of both C/EBPε^32^ (amino acid 121) and C/EBPε^27^ (amino acid 92) were generated by site directed mutagenesis (Figure 6D). The target lysines were converted to either arginine (R) or alanine (A), and the mutant constructs tested for their ability to inhibit GATA-1 transactivation of the MBP1-P2 promoter in comparison to wild type C/EBPε^27^ and C/EBPε^32^ (Figure 6E). If sumoylation contributes to the ability of C/EBPε^27^ to repress GATA-1 transactivation of the MBP1-P2 promoter, mutation of the target VKEEP lysine residue would be expected to eliminate its repressor activity, and for C/EBPε^32^, might allow for increased activity for its target genes, and/or synergy with GATA-1, as we have previously shown for GATA-1 and C/EBPβ [17], and C/EBPα (Du and Ackerman, unpublished results). As shown in Figure 6E, the K→R and K→A point mutations in the VKEEP sequences of C/EBPε^27^ and C/EBPε^32^ did not relieve repression mediated by their RDI domain, nor relieve C/EBPε^27^ antagonism of GATA-1. Together, these findings indicate that despite our observation that C/EBPε^27^ is sumoylated in vivo in eosinophilic cells, its repressor activity for GATA-1 does not require this post-translational modification, in marked contrast to murine C/EBPε [19].

### 2.6. Deletion of the DNA Binding Domain of C/EBPε^27^ Only Partially Relieves Repressor Activity for GATA-1

**Figure 7:**
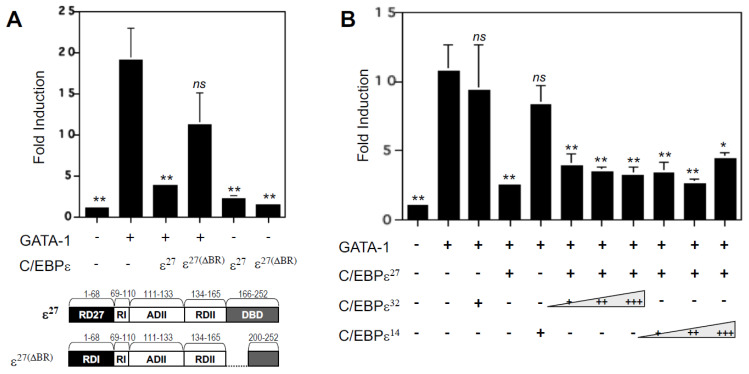
Binding of C/EBPε^27^ to DNA is not required for its repression of GATA-1. (**A**) Structures of wild type C/EBPε^27^ and the C/EBPε^27^ ΔBR mutant construct (deletion of the basic region of the DNA binding domain, amino acids 166–199) are shown. Fold induction of MBP1-P2 promoter luciferase activity is shown for CV-1 cells co-transfected with GATA-1, C/EBPε^27^ and the C/EBPε^ΔBR^ mutant as indicated. Deletion of the basic region of the DNA binding domain of C/EBPε^27^ (ΔBR) partially relieves its repression of GATA-1 transactivation of the MBP1-P2 promoter. (**B**) Co-transfection of CV-1 cells with expression vectors for the C/EBPε^32^ and C/EBPε^14^ isoforms does not compete with (block) the ability of C/EBPε^27^ to function as a repressor of GATA-1 transactivation of the MBP1-P2 promoter. * *p* < 0.05; ** *p* < 0.01; *ns*, not significant, compared to GATA-1 alone.

We previously reported that C/EBPε^27^ and GATA-1 physically interact in vivo in eosinophil myelocyte cell lines such as AML14.3D10 using co-immunoprecipitation assays [11]. To further elucidate the mechanism by which C/EBPε^27^ attenuates GATA-1 transcriptional activity, we determined whether its inhibitory activity requires DNA binding to the C/EBP site immediately upstream of the dual GATA site in the MBP promoter (Figure 1A), or whether protein–protein interaction is sufficient for its antagonism. As shown in Figure 7A, deletion of the DNA-binding domain (amino acids 166–169) of the basic region of C/EBPε^27^ (ε^ΔBR^), a mutation which leaves the leucine zipper dimerization domain intact, only partially abrogated its repressor activity for GATA-1 compared to wild type C/EBPε^27^. As shown in Figure 1A, a functional C/EBP binding site required for MBP1-P2 promoter activity [17] is present immediately upstream of the high affinity dual (double) GATA site. These results suggest that the binding of C/EBPε^27^ to this C/EBP site may foster (enhance) its physical interaction with GATA-1, leading to antagonism of GATA-1, but that the DNA-bound intermediate is not required for their protein–protein interaction to occur. Since mutation of the C/EBP site leads to complete inactivation of the MBP1-P2 promoter in eosinophilic cell lines and in transactivation experiments in heterologous cell lines [11,17], mutation of this site could not be directly utilized to address the importance of C/EBPε^27^ DNA binding in its repressor interaction with GATA-1. To further address the role of C/EBPε^27^ DNA binding in this interaction, we instead tested the ability of the other C/EBPε isoforms (C/EBPε^32^ and C/EBPε^14^) by binding to the upstream C/EBP site and/or heterodimerizing with C/EBPε^27^, to block its repressor activity for GATA-1. However, neither of the other C/EBPε isoforms (analyzed in dose-response experiments) could relieve C/EBPε^27^ repression of GATA-1 (Figure 7B). Taken together, these results indicate that DNA binding by C/EBPε^27^ is not a requirement for its ability to physically interact with and antagonize GATA-1, thus allowing C/EBPε^27^ to attenuate GATA-1 activity under all potential conditions in the regulation of MBP1 gene transcription.

## 3. Discussion

In the current study, using the HIV Tat transduction peptide [29] to target a Tat-C/EBPε^27^ fusion protein to the eosinophil’s nucleus, we show that C/EBPε^27^ potently inhibits endogenous MBP1 gene transcription in an eosinophil myelocyte cell line (AML14.3D10), demonstrating its activity as a repressor in vivo. Our results indicate that several repression domains in C/EBPε^27^ contribute to its attenuation of GATA-1 transactivation, particularly its unique N-terminal domain. Although C/EBPε^32^ and C/EBPε^27^ are both sumoylated, the addition of SUMO-1 does not appear to affect the ability of either isoform to regulate the MBP1 gene. Co-transfection of a SUMO-1 expression vector with either C/EBPε^32^ or C/EBPε^27^ in transactivation assays with or without GATA-1, as well as point mutations generated in the sumoylated lysine residue of the VKEEP SUMO consensus site found in both isoforms, does not alter the activity of either isoform for GATA-1 or the MBP1-P2 promoter. We conclude that sumoylation of C/EBPε^27^ does not play a role in the repressor activity of this isoform, nor convert full length C/EBPε^32^ into a repressor. Importantly, contributions of two repressor domains present in C/EBPε^27^, its unique 68 amino acid N-terminal region, and a conserved segment of the RDI core domain shared with C/EBPε^32^, is required for this transcription factor to antagonize the potent transcriptional activity of GATA-1. Finally, deletion of the DNA-binding basic region of C/EBPε^27^ partially relieves its repressor activity, indicating that GATA-1 antagonism is enhanced by, but does not require, DNA binding to a C/EBP site immediately upstream of the high affinity double GATA-1 binding site in this gene.

In the mouse, C/EBPε is required for granulocyte (both eosinophil and neutrophil) terminal differentiation, secondary granule gene expression, and regulation of this process, with a block in the promyelocyte to myelocyte transition demonstrated in two different knockout (null) strains [5,6,10]. Of note, murine C/EBPε is expressed as only two isoforms, generally equivalent in size, structure and function to the human 32kD and 30kD isoforms [4,19], whereas shorter orthologs of the human C/EBPε^27^ and C/EBPε^14^ isoforms are completely lacking [10]. For human myeloid progenitors, expression of C/EBPε^32/30^ and the shorter C/EBPε^27^ and C/EBPε^14^ isoforms is developmentally regulated during both neutrophil [1,3] and eosinophil [12] differentiation. We previously reported a role for the C/EBPε^27^ isoform as a potent antagonist of GATA-1 activity for the eosinophil MBP1-P2 promoter [11] and showed that GATA-1 physically interacts with the C/EBPε isoforms including C/EBPε^27^ in eosinophil myelocytes (the AML14.3D10 cell line). In the current studies, we extended these findings, demonstrating by ChIP analyses that both C/EBPε and GATA-1 occupy the eosinophil MBP1-P2 promoter in AML14.3D10 eosinophil myelocytes that actively express MBP1 mRNA and protein [23,24]. Importantly, using the HIV Tat protein transduction system for high efficiency targeting of proteins to both the cytosol and nucleus of proliferating and non-dividing cells [29,30,31], we generated a Tat-C/EBPε^27^ fusion protein and used it to show that C/EBPε^27^ is a potent inhibitor in vivo of endogenous MBP1 gene transcription. In addition to confirming the suggested repressor activities for both the C/EBPε^27^ and C/EBPε^14^ isoforms, this approach extends the utility of using HIV Tat protein transduction to target transcription factors directly to the nucleus for structure-function studies of their activities in regulating endogenous gene transcription [32].

We used TAT-C/EBPε^27^ and TAT-C/EBPε^14^ fusion proteins to efficiently transduce > 99% of AML14.3D10 eosinophil myelocytes and monitored their effects on endogenous MBP1 gene expression. The TAT-C/EBPε^27^ fusion protein was efficiently transduced and translocated into the nucleus where it was able to inhibit, in a dose-dependent manner, endogenous MBP1 gene expression by up to ~50% (Figure 3), but higher concentrations did not decrease steady state levels of MBP1 mRNA further. One possible explanation for the plateau of inhibition at ~50% may be the half-life of MBP1 mRNA within the short time frame (8 h) for these experiments, and that the Tat-fusion protein may reach an equilibrium between nuclear, cytosolic and extracellular compartments., since the transduced Tat-fusion protein is capable of moving in and out of the nucleus and back across the plasma membrane until an equilibrium is reached. Our initial experiments using transduction for 24 h (Figure 3) suggested even greater levels of inhibition of ~70% and may well represent significantly greater inhibition (up to 100%) of new mRNA transcription.

We also tested a TAT-C/EBPε^14^ fusion protein hypothesized to function as a natural dominant negative repressor of other C/EBP family members, due to its lack of a transactivation domain and possession of basic DNA binding and leucine zipper dimerization domains common to the other C/EBPε isoforms and other C/EBPs [1]. However, the C/EBPε^14^ isoform does not inhibit GATA-1 activity in our MBP1-P2 reporter assays [11] but does inhibit C/EBPα and C/EBPβ transactivation of the MBP P2 promoter [11] and would therefore be predicted to inhibit endogenous MBP1 gene expression. However, TAT-C/EBPε^14^ was only able to inhibit MBP1 mRNA expression by ~20–25% under identical transduction conditions used for TAT-C/EBPε^27^ (Figure 4). Differences between these C/EBPε isoforms may be due to differences in the mechanisms by which they repress MBP1-P2 promoter activity, C/EBPε^27^ antagonizing GATA-1 activity and other C/EBPs, while C/EBPε^14^ competes only with C/EBPε^32^ and the other C/EBPs (i.e., C/EBPα and β), both of which we showed exist as homo- and heterodimers with C/EBPε in eosinophil myelocytes and are inhibited by C/EBPε^14^ and C/EBPε^27^ [11]. Thus, we suggest the higher repressor activity of TAT-C/EBPε^27^ may be due to its ability to interact with both GATA-1 and other C/EBPs, while C/EBPε^14^ is restricted to solely antagonizing other C/EBPs through heterodimerization or DNA binding as a homodimer. These results support negative regulatory roles for both C/EBPε^27^ and C/EBP^14^ in eosinophil gene transcription and highlight the utility of using TAT-fusion proteins for in vivo studies of gene regulation in difficult to transfect myeloid and other cells.

We also performed an extensive structure-function analysis of the C/EBPε^27^ isoform to map its GATA-1 repressor domains, with comparisons to both the full-length C/EBPε^32^ activator isoform, and the shorter C/EBPε^14^ putative repressor isoform. Results showed that C/EBPε^27^ repression of GATA-1 activity is mediated in part by its unique N-terminus combined with the previously identified RDI core domain (shared with C/EBPε^32^). Of note, this repressor activity does not require, but was enhanced by, DNA binding of C/EBPε^27^, likely to the C/EBP site immediately upstream and adjacent to the double GATA site in the MBP1-P2 promoter, since we previously showed that this site is an absolute requirement for activity of this promoter [11], and is responsible for synergistic activation by C/EBPβ and GATA-1 [17]. We also showed that the repressor activity of C/EBPε^27^ is independent of sumoylation of the “VKEEP” consensus sumoylation site in its RDI core domain, in marked contrast to murine C/EBPε for which sumoylation of the “VKEEP” sequence is a prerequisite for RDI domain repressor activity [19,33]. Additionally, we defined the unique N-terminus of C/EBPε^27^, the distinct 68 amino acid sequence (RD27) not shared with the other C/EBPε isoforms or C/EBP family members [10], as the minimum domain required for antagonism of GATA-1; the RD27 domain alone has the capacity to convert the C/EBPε^32^ transcriptional activator isoform into a repressor of GATA-1. Our previously reported findings for the C/EBPε^14^ isoform using co-transactivation assays [11], and the current studies using Tat-mediated transduction of eosinophil myelocytes (Figure 4), confirm it as a naturally expressed, dominant negative transcriptional repressor. These observations support our prior hypothesis that one of the likely roles of the C/EBPε^27^ and C/EBPε^14^ isoforms may be to down-regulate and turn off (repress) expression of secondary granule protein genes such as MBP1 during eosinophil terminal differentiation (see Figure 11 in reference [11]), since these genes are ultimately silenced in the mature cell [34].

We have used the MBP1-P2 promoter as a model for GATA-1-regulated gene expression in the eosinophil lineage to provide the first structure-function elucidation of the repressor domains of C/EBPε^27^ responsible for its ability to potently inhibit GATA-1-mediated gene transcription. Mutational analyses of C/EBPε^27^ identify both its unique N-terminal 68 amino acid domain (RD27) and its highly conserved “VKEEP” segment within the previously identified RDI repressor domain also found in C/EBPε^32^, as key contributors to the ability of C/EBPε^27^ to repress GATA-1-mediated transactivation. The role of sumoylation was explored further using site-directed mutagenesis of the target lysine in the conserved “VKEEP” SUMO sites in both C/EBPε^27^ and C/EBPε^32^, mutations that block their sumoylation. However, mutation of the target lysines had no effect on the ability of C/EBPε^27^ to block GATA-1 activity, nor did it convert C/EBPε^32^ into an activator (or repressor) of the MBP1-P2 promoter in the absence or presence of GATA-1. Finally, since we have detected sumoylated C/EBPε in the AML14.3D10 eosinophil line, which expresses all the human C/EBPε isoforms, an in-vivo role for sumoylation of C/EBPε may still be possible. Of interest, Subramanian and colleagues identified a conserved synergy control (SC) motif within the negative regulatory domains of C/EBPα and other transcription factors that regulates their synergistic interactions [20]. A K159→R substitution within this SC motif did not alter C/EBPα transcriptional activity from a single C/EBP site, but enhanced transactivation from compound C/EBP sites. This SC motif overlaps with the consensus SUMO modification site in C/EBPα, which is modified by both SUMO-1 and SUMO-3 in vitro and in vivo by the E2 SUMO-conjugating enzyme Ubc9 [20]. These findings suggest that sumoylation of SC motifs provides a mechanism to rapidly control higher order interactions among transcription factors that may be a general mechanism to limit transcriptional synergy. The MBP1-P2 promoter contains an additional C/EBP site further downstream that might participate in these sorts of interactions.

The only mutant of C/EBPε^27^ that was not inhibitory to GATA-1 activation of the MBP1-P2 promoter is the “natural” mutant of both C/EBPε^32^ and C/EBPε^27^, ε^Δ1–123^, which is equivalent to the shortest C/EBPε^14^ isoform. Also, the ε^Δ2–68^ construct is a mutant not only C/EBPε^27^, but of C/EBPε^32^ as well, since C/EBPε^32^ lacks this repressor sequence, highlighting the anti-repressor role of the N-terminus of C/EBPε^32^ in attenuating the activity of the RDI repressor domain, and thus the potential of C/EBPε^32^ to act as a repressor as previously reported [2] (though in the current studies, not a transcriptional activator either). The mutants of human C/EBPε, whether derived solely from C/EBPε^27^ or C/EBPε^32^, illustrate the continuum of activities instructed by the individual repressor (RDI, RDII, RD27) versus activation domains of the isoforms characterized here, and previously by others [2,19,33,35].

As eosinophil-committed progenitors differentiate, stage-specific gene expression is controlled by various temporally regulated and tissue specific transcription factors. For granulocytes in general, hematopoietic-specific C/EBPε plays an additional role in the exit from cell cycle at the promyelocyte to myelocyte transition, as well as terminal differentiation of the granulocyte lineages, including the eosinophil lineage [36]. It is possible that there is a distinction between the roles of C/EBPε^32^ and C/EBPε^27^ in regulation of their target genes. For C/EBPε^27^, its principal role may be to attenuate GATA-1 activity during the final stages of eosinophil terminal differentiation, allowing MBP1 and other secondary granule genes such as MBP2, EPX and the eosinophil ribonucleases EDN (RNase2) and ECP (RNase3) to be silenced through antagonizing this potent activator, since these genes are no longer expressed in the mature blood eosinophil [34]. Of note, Mack and colleagues [37] reported that Trib1, a regulator of granulocyte development, functions in promoting development of eosinophils by targeting C/EBPα for protein degradation, and C/EBPα has been shown to bind to an upstream 6kb enhancer site of the C/EBPε gene, thus promoting C/EBPε transcription [38]. Trib1-induced degradation of C/EBPα would thus reduce levels of C/EBPε. Since it is known that Trib1 expression increases eosinophil lineage identity, it is likely that expression of Trib1 implies reduction in levels of, including but not necessarily limited to, the C/EBPε^27^ isoform, as our own studies have shown that C/EBPε^27^ reduces expression of the eosinophil secondary granule protein genes [12]. Thus, expression of Trib1 would allow for eosinophil granule proteins to be expressed in early eosinophilic development. This supports our hypothesis that C/EBPε^27^ is important in the final stages of eosinophil development for silencing genes no longer active in mature eosinophils, as earlier activation of C/EBPε, as in the case of Trib1 deletion, leads to development of cells with more neutrophilic characteristics, this due to earlier silencing of eosinophil-specific genes resulting from the earlier presence of C/EBPε^27^ [37]. This in concert with additional functions identified for C/EBPε^32^, which has been shown to transcriptionally activate the Mad1 gene and hence turn on expression of this transcriptional repressor involved in cell cycle arrest necessary for eosinophil terminal differentiation [36]. Nakajima and colleagues [39] mapped the N-terminal activation domain of full-length C/EBPε as being required for murine granulocyte progenitor cell cycle arrest, functional maturation, and apoptosis during granulocyte differentiation, showing that C/EBPε up-regulates p27 and down-regulates cyclins/cdks to induce growth arrest during this process. In addition, C/EBPε was shown to induce apoptosis by down-regulating the anti-apoptotic bcl-2 and bcl-x proteins, effects likewise mediated by its N-terminal activation domain, which was also required for induction of neutrophil secondary granule protein genes [39]. It is possible that other genes activated or repressed during granulocyte differentiation, as with Mad1 or bcl-2/bcl-x, may be regulated by full length C/EBPε, but it is not known whether this is also true for the human C/EBPε^27^ or C/EBPε^14^ repressor isoforms.

In summary, we characterized a novel role for the unique N-terminus of C/EBPε^27^ (RD27) for repression of GATA-1 transactivation of the eosinophil’s MBP1-P2 promoter [11]. Although we previously suggested that C/EBPε^27^-GATA-1 protein–protein interaction is necessary and sufficient for this transcriptional antagonism [11], our current results suggest this inhibitory effect may be mediated in part through a C/EBPε^27^ DNA-bound component. Thus, we suggest C/EBPε^27^ can interact with GATA-1 strictly through a solution protein–protein interaction, but that recruitment of C/EBPε^27^ to its consensus site immediately upstream of the high affinity double GATA-1 site in the MBP1-P2 promoter may promote enhanced access of C/EBPε^27^ homodimers to GATA-1 for repressor activity. Experiments to elaborate on this theme are warranted to determine whether antagonism of C/EBPε^27^ for GATA-1 holds true for other GATA-1-regulated eosinophil or other myeloid genes that contain proximal C/EBP sites.

## 4. Materials and Methods

### 4.1. Cell Lines and Transfections

CV-1 and COS-7 cell lines were maintained in Dulbecco’s modified Eagle’s Medium (Invitrogen) supplemented with 10% fetal bovine serum (GIBCO, Invitrogen, Waltham, MA, USA). COS-7 cells were used for protein expression experiments. Cells plated on 10 cm tissue culture plates were transfected with 5µg of each expression construct using FUGENE 6 (Roche, San Francisco, CA, USA) as the transfection reagent according to manufacturer’s guidelines. CV-1 cells grown in 6-well plates were used for transactivation assays as previously described [11]. The AML14.3D10 eosinophil myelocyte cell line was cultured and transfected by electroporation as previously described [11], and 1.5 × 10^7^ cells were used for each transfection.

### 4.2. Plasmid Constructs

The SUMO-1 and mutant SUMO-1 expression vectors (pCMV-FLAG-SUMO-1) were kindly provided by Dr. Giuseppina Nucifora (University of Illinois at Chicago). Expression vectors for the various C/EBPε isoforms were kindly provided by Drs. Kleanthis Xanthopoulos and Julie Lekstrom-Himes (NIH) and have been previously described [1,11]. Site-directed mutagenesis was performed using the Exsite™ and QuikChange™ mutagenesis kits from Stratagene following manufacturers’ instructions with the exception of using *Pfu* Ultra (Stratagene, La Jolla, CA, USA) as the DNA polymerase for the Exsite™ kit. The following primers (5′-3′) were used to create the indicated deletions and point mutations for pcDNA-C/EBPε^27^: For Δ2–68—Forward: GACAGGAAG GCGCTGGGGCCTGGCATCTAC; Reverse: CATTTGCTAAGCTTGGGTCTCCCTATAGTG; For Δ69–100—Forward: AGCCGAGCTGCCAG CCGAGGC; Reverse: TCCTCCCTTGACACGC CTTCCTCTGGC; For Δ69–123—Forward: ATG CACCTGCCCCCAACTCTGGCAG; Reverse: TCCTCCCTTGACACGCCTTCCTCTGGC; For Δ69–165—Forward: AAFGGCAAGAAGGCA GTGAACAAAGATAGC; Reverse: TCCTCCCT TGACACGCCTTCCTCT; For ΔBR—Forward: ATTCTGGAGACGCAGCAGAAGGTGCTGGAG; Reverse: GTGTAAGGGGCCAGCCGGGGAG G; For K→R mutant (for both pCDNA-C/EBPε^32^ and pcDNA-C/EBPε^27^)—Forward: CTGTGGCGG TGAGGGAGGAGCCCCG; Reverse: CGGGGCT CCTCCCTCACCGCCACAG; For K→A mutant—Forward: GTGGCGGTGGCGGAGGAGCCCC GGG; Reverse: CCCGGGGCTCCTCCGCCACC GCCAC; Primers for generation of the C/EBPε^32^ fusion (N-terminal 68 amino acids of C/EBPε^27^ added to the N-terminus of C/EBPε^32^): N-terminal primers for C/EBPε^27^—Forward: (AscI restriction site) TTGGCGCGCCCCGGCCATGAGCATGCT CTGGAGCAC; Reverse: TCCTCCCTTGACAC GCCTT; Primers for pcDNA-C/EBPε^32^ used for insertion of this DNA segment of C/EBPε^27^ into pcDNA-C/EBPε^32^—Forward: ATGTCCCACGGG ACCTACTA; Reverse: TTGGCGCGCCGGCCG GCCCGCCCCCTCG.

### 4.3. Chromatin Immunoprecipitation (ChIP)

ChIP assays were performed using methods adapted from Wells et al. [40] and Takahashi et al. [41]. All steps were performed on ice or at 4 °C. AML14.3D10 eosinophil myelocytes (1–2 × 10^7^ cells) were cross-linked with formaldehyde at a final concentration of 1% (*W*/*V*) for 10 min. at room temperature. Reactions were stopped by the addition of glycine to a final concentration of 125 µM for 5 min. at room temperature. Cells were washed in cold PBS, centrifuged, and allowed to swell in RSB buffer (3 mM MgCl_2_, 10 mM NaCl, 10 mM Tris (pH7.4) and 0.1% NP-40 (Roche), with protease inhibitors including 1 mM PMSF and a Roche Complete Inhibitor Tablet) on ice for 10 min. Nuclei were collected by centrifugation using a microcentrifuge (Beckman Coulter, Brea, CA, USA) at 3500 rpm for 10 min. at 4 °C, and lysed in nuclear lysis buffer (1% SDS, 1.1% Triton-X-100, 1.2 mM EDTA, 20 mM Tris (pH 8.1), 167 mM NaCl, with protease inhibitors as above) on ice for 10 min. The nuclear lysates were sonicated using a Branson Model 450 Sonicator (Branson Ultrasonics Corporation, Danbury, CT, USA) for 4 min at a 60% duty cycle and power level 4 setting with a tapered microtip until the average chromatin fragment size was 600–700 bp. The sonicated nuclear lysates were then diluted 1:5 with immunoprecipitation (IP) dilution buffer (0.01% SDS, 1.1% Triton X-100, 1.2 mM EDTA, 20 mM Tris (pH 8.1), 167 mM NaCl, with protease inhibitors as above). The nuclear lysates were pre-cleared with 100 µL pre-blocked Protein G-Sepharose (Amersham/GE Healthcare, Chicago, IL, USA) (blocked with 1 µg/µL BSA and sonicated salmon sperm DNA in IP dilution buffer) for 8 h. Nuclear lysates were then immunoprecipitated with 2 µg of normal non-immune IgG isotype control or specific antibody to the transcription factors of interest overnight with rotation at 4 °C. Antigen-antibody complexes were immunoprecipitated using 20 µL of blocked Protein G-Sepharose, and the beads washed 7 times with RIPA buffer. Protein/DNA cross-links were reversed by the addition of 100 µL of TE to the washed pellets along with RNase A and Proteinase K, both at final concentrations of 10 µg per sample, followed by incubation at 55 °C for 3 h. and overnight at 65 °C. The samples were then subjected to multiple phenol/chloroform extractions to remove nuclear proteins, and the DNA was finally ethanol-precipitated and used for PCR amplifications of the MBP1-P2 promoter or control β-actin gene sequences of interest. Primers used for the MBP1-P2 promoter 280bp product were: Forward—AAAAGCACC CAAGGCGATTC (bp −253 to −234); Reverse—ATCTTCCCAAAGCCCAGGTCCTTC (bp +4 to +27); Primers for the human β-actin gene (266bp product) were: Forward—TTCTCACTGGTTCTCTCTTCTG CC; Reverse—TTGGGATGGGGAGTCTGTTCAG. Immunoprecipitation assays were performed as previously described for AML14.3D10 eosinophil and COS-7 cell lysates [11]. For detection of sumoylated proteins, 50 mM *N*-ethylmaleimide was added as an isopeptidase inhibitor. For detection of SUMO-1, either an anti-FLAG-HRP antibody (M2, Sigma Aldrich, St. louis, MI, USA) or a mouse anti-SUMO-1 monoclonal antibody (Santa Cruz Biotechnology, Dallas, TX, USA) was used. For IP of C/EBPε, an anti-C/EBPε polyclonal rabbit antibody was used (Santa Cruz Biotechnology). For the GATA-1 ChIP assays, a polyclonal goat anti-GATA-1 was used (Santa Cruz Biotechnology). For all immuno-precipitations, 1 µg of total IgG antibody was used, and 1µg/mL was used for detection of proteins by Western blotting.

### 4.4. Tat-C/EBPε Fusion Proteins and Cell Transductions

The vectors for generation of the TAT-C/EBPε fusion protein constructs (pTAT-HA) and the control TAT-GFP expression construct were kindly provided by Drs. Paul Bertics and David Hall, University of Wisconsin-Madison [30]. The C/EBPε^27^ and C/EBPε^14^ expression vectors have been described previously [1,10] and were used to subclone the C/EBPε isoform cDNAs into the pTAT-HA vector using the KpnI and EcoR1 restriction sites. Purification of the TAT-fusion proteins was done by nickel affinity chromatography (Qiagen, Germantown, MD, USA) using the N-terminal His-tag present in the TAT-fusion proteins of interest expressed from the pTAT-HA vector constructs as previously described [30,42]. Protein transductions of the AML14.3D10 eosinophil myelocyte cell line with the various C/EBPε isoform and control fusion proteins was performed as previously described for transduction of purified peripheral blood eosinophils [30,42]. Negative control TAT-fusion proteins included purified PG-cTAT [25] containing the IgG binding domain of streptococcal protein G fused to the c-terminus of the TAT peptide (kindly provided by Susanna Hakansson and Dr. Michael Caffrey, University of Illinois at Chicago) or TAT-GFP. Transactivation assays of TAT-fusion protein transduced cells were performed as previously described [11]. Real Time RT-PCR for quantitation of MBP1 and GAPDH mRNA expression used TaqMan™ probes (6-FAM-CAC AGG CTC GGG TCG CTG CA-TAMRA) designed using Primer Express software (Applied Biosystems. Waltham, MA). Primers for the MBP1 gene were: Forward—5′-AAGTCTGGATTGGAGGCAGG A-3′; Reverse—5′-CGTCAACCCACTGAAAGCG T-3′. Real Time RT-PCR reactions were carried out using Applied Biosystems Taqman One Step RT-PCR master mix reagents and Taqman GAPDH control reagents. All reactions used the Perkin Elmer/Applied Biosystems ABI Prism 7700 Sequence Detection system. Cycling conditions were: 48 °C for 30 min followed by 95 °C for 10 min; 95 °C for 15 s followed by 56 °C for 1 min for 40 cycles. Confocal microscopy was performed in the Confocal Microscopy Facility of the Research Resources Center of the University of Illinois at Chicago.

### 4.5. Statistical Analysis

Unpaired Student’s *t*-test was used to compare means. Differences were considered statistically significant at *p* < 0.05. Error bars represent ±SD. Statistical analyses were done using Prism 8 (GraphPad Software, San Diego, CA, USA).

## 5. Conclusions

C/EBPε^27^ antagonism of the transcriptional activity of GATA-1 in the human eosinophil lineage during differentiation is mediated by a unique N-terminal repression domain, does not require sumoylation of this domain, and occurs independently of, but is enhanced by, DNA binding.

## Data Availability

The data presented in this study are available on request from the corresponding author.

## Supplementary Materials

The following are available online at https://www.mdpi.com/article/10.3390/ijms222312689/s1.

## Abbreviations

C/EBP: CCAAT/Enhancer-binding protein; C/EBPε32, C/EBPε27, and C/EBPε14, CCAAT/Enhancer-binding protein epsilon 32kD, 27kD, and 14kD isoforms, respectively; MBP1, eosinophil granule major basic protein-1; BR, basic region (DNA binding domain of C/EBPε); IP, immunoprecipitation; ChIP, chromatin immunoprecipitation.

## References

[1] Yamanaka R., Kim G.D., Radomska H.S., Lekstrom-Himes J., Smith L.T., Antonson P., Tenen D.G., Xanthopoulos K.G. (1997). CCAAT/enhancer binding protein epsilon is preferentially up-regulated during granulocytic differentiation and its functional versatility is determined by alternative use of promoters and differential splicing. Proc. Natl. Acad. Sci. USA.

[2] Williamson E.A., Xu H.N., Gombart A.F., Verbeek W., Chumakov A.M., Friedman A.D., Koeffler H.P. (1998). Identification of transcriptional activation and repression domains in human CCAAT/enhancer-binding protein epsilon. J. Biol. Chem..

[3] Morosetti R., Park D.J., Chumakov A.M., Grillier I., Shiohara M., Gombart A.F., Nakamaki T., Weinberg K., Koeffler H.P. (1997). A novel, myeloid transcription factor, C/EBP epsilon, is upregulated during granulocytic, but not monocytic, differentiation. Blood.

[4] Williams S.C., Du Y., Schwartz R.C., Weiler S.R., Ortiz M., Keller J.R., Johnson P.F. (1998). C/EBPepsilon is a myeloid-specific activator of cytokine, chemokine, and macrophage-colony-stimulating factor receptor genes. J. Biol. Chem..

[5] Yamanaka R., Barlow C., Lekstrom-Himes J., Castilla L.H., Liu P.P., Eckhaus M., Decker T., Wynshaw-Boris A., Xanthopoulos K.G. (1997). Impaired granulopoiesis, myelodysplasia, and early lethality in CCAAT/enhancer binding protein epsilon-deficient mice. Proc. Natl. Acad. Sci. USA.

[6] Verbeek W., Wachter M., Lekstrom-Himes J., Koeffler H.P. (2001). C/EBPε−/− mice: Increased rate of myeloid proliferation and apoptosis. Leukemia.

[7] Lekstrom-Himes J.A., Dorman S.E., Kopar P., Holland S.M., Gallin J.I. (1999). Neutrophil-specific granule deficiency results from a novel mutation with loss of function of the transcription factor CCAAT/enhancer binding protein epsilon. J. Exp. Med..

[8] Gombart A.F., Shiohara M., Kwok S.H., Agematsu K., Komiyama A., Koeffler H.P. (2001). Neutrophil-specific granule deficiency: Homozygous recessive inheritance of a frameshift mutation in the gene encoding transcription factor CCAAT/enhancer binding protein–epsilon. Blood.

[9] Rosenberg H.F., Gallin J.I. (1993). Neutrophil-specific granule deficiency includes eosinophils. Blood.

[10] Lekstrom-Himes J.A. (2001). The role of C/EBP(epsilon) in the terminal stages of granulocyte differentiation. Stem. Cells.

[11] Du J., Stankiewicz M.J., Liu Y., Xi Q., Schmitz J.E., Lekstrom-Himes J.A., Ackerman S.J. (2002). Novel combinatorial interactions of GATA-1, PU.1, and C/EBPepsilon isoforms regulate transcription of the gene encoding eosinophil granule major basic protein. J. Biol. Chem..

[12] Bedi R., Du J., Sharma A.K., Gomes I., Ackerman S.J. (2009). Human C/EBP-epsilon activator and repressor isoforms differentially reprogram myeloid lineage commitment and differentiation. Blood.

[13] Hirasawa R., Shimizu R., Takahashi S., Osawa M., Takayanagi S., Kato Y., Onodera M., Minegishi N., Yamamoto M., Fukao K. (2002). Essential and instructive roles of GATA factors in eosinophil development. J. Exp. Med..

[14] Yu C., Cantor A.B., Yang H., Browne C., Wells R.A., Fujiwara Y., Orkin S.H. (2002). Targeted deletion of a high-affinity GATA-binding site in the GATA-1 promoter leads to selective loss of the eosinophil lineage in vivo. J. Exp. Med..

[15] Humbles A.A., Lloyd C.M., McMillan S.J., Friend D.S., Xanthou G., McKenna E.E., Ghiran S., Gerard N.P., Yu C., Orkin S.H. (2004). A critical role for eosinophils in allergic airways remodeling. Science.

[16] Yamaguchi Y., Ackerman S.J., Minegishi N., Takiguchi M., Yamamoto M., Suda T. (1998). Mechanisms of transcription in eosinophils: GATA-1, but not GATA-2, transactivates the promoter of the eosinophil granule major basic protein gene. Blood.

[17] Yamaguchi Y., Nishio H., Kishi K., Ackerman S.J., Suda T. (1999). C/EBPbeta and GATA-1 synergistically regulate activity of the eosinophil granule major basic protein promoter: Implication for C/EBPbeta activity in eosinophil gene expression. Blood.

[18] McNagny K., Graf T. (2002). Making eosinophils through subtle shifts in transcription factor expression. J. Exp. Med..

[19] Kim J., Cantwell C.A., Johnson P.F., Pfarr C.M., Williams S.C. (2002). Transcriptional activity of CCAAT/enhancer-binding proteins is controlled by a conserved inhibitory domain that is a target for sumoylation. J. Biol. Chem..

[20] Subramanian L., Benson M.D., Iniguez-Lluhi J.A. (2003). A synergy control motif within the attenuator domain of CCAAT/enhancer-binding protein alpha inhibits transcriptional synergy through its PIASy-enhanced modification by SUMO-1 or SUMO-3. J. Biol. Chem..

[21] Eaton E.M., Sealy L. (2003). Modification of CCAAT/enhancer-binding protein-beta by the small ubiquitin-like modifier (SUMO) family members, SUMO-2 and SUMO-3. J. Biol. Chem..

[22] Verger A., Perdomo J., Crossley M. (2003). Modification with SUMO. A role in transcriptional regulation. EMBO Rep..

[23] Paul C.C., Mahrer S., Tolbert M., Elbert B.L., Wong I., Ackerman S.J., Baumann M.A. (1995). Changing the differentiation program of hematopoietic cells: Retinoic acid-induced shift of eosinophil-committed cells to neutrophils. Blood.

[24] Baumann M.A., Paul C.C. (1998). The AML14 and AML14.3D10 cell lines: A long-overdue model for the study of eosinophils and more. Stem Cells.

[25] Hakansson S., Jacobs A., Caffrey M. (2001). Heparin binding by the HIV-1 tat protein transduction domain. Protein Sci..

[26] Lekstrom-Himes J., Xanthopoulos K.G. (1998). Biological role of the CCAAT/Enhancer-binding protein family of transcription factors. J. Biol. Chem..

[27] Angerer N.D., Du Y., Nalbant D., Williams S.C. (1999). A short conserved motif is required for repressor domain function in the myeloid-specific transcription factor CCAAT/enhancer-binding protein epsilon. J. Biol. Chem..

[28] Ross S., Best J.L., Zon L.I., Gill G. (2002). SUMO-1 modification represses Sp3 transcriptional activation and modulates its subnuclear localization. Mol. Cell.

[29] Wadia J.S., Dowdy S.F. (2002). Protein transduction technology. Curr. Opin. Biotechnol..

[30] Hall D.J., Cui J., Bates M.E., Stout B.A., Koenderman L., Coffer P.J., Bertics P.J. (2001). Transduction of a dominant-negative H-Ras into human eosinophils attenuates extracellular signal-regulated kinase activation and interleukin-5-mediated cell viability. Blood.

[31] Nagahara H., Vocero-Akbani A.M., Snyder E.L., Ho A., Latham D.G., Lissy N.A., Becker-Hapak M., Ezhevsky S.A., Dowdy S.F. (1998). Transduction of full-length TAT fusion proteins into mammalian cells: TAT-p27Kip1 induces cell migration. Nat. Med..

[32] Patruno M., Melotti L., Gomiero C., Sacchetto R., Topel O., Martinello T. (2017). A mini-review of TAT-MyoD fused proteins: State of the art and problems to solve. Eur. J. Transl. Myol..

[33] Kim J., Sharma S., Li Y., Cobos E., Palvimo J.J., Williams S.C. (2005). Repression and coactivation of CCAAT/enhancer-binding protein epsilon by sumoylation and protein inhibitor of activated STATx proteins. J. Biol. Chem..

[34] Gruart V., Truong M.J., Plumas J., Zandecki M., Kusnierz J.P., Prin L., Vinatier D., Capron A., Capron M. (1992). Decreased expression of eosinophil peroxidase and major basic protein messenger RNAs during eosinophil maturation. Blood.

[35] Tang J.G., Koeffler H.P. (2001). Structural and functional studies of CCAAT/enhancer-binding protein epsilon. J. Biol. Chem..

[36] Walkley C.R., Purton L.E., Snelling H.J., Yuan Y.D., Nakajima H., Chambon P., Chandraratna R.A., McArthur G.A. (2004). Identification of the molecular requirements for an RAR alpha-mediated cell cycle arrest during granulocytic differentiation. Blood.

[37] Mack E.A., Stein S.J., Rome K.S., Xu L., Wertheim G.B., Melo R.C.N., Pear W.S. (2019). Trib1 regulates eosinophil lineage commitment and identity by restraining the neutrophil program. Blood.

[38] Shyamsunder P., Shanmugasundaram M., Mayakonda A., Dakle P., Teoh W.W., Han L., Kanojia D., Lim M.C., Fullwood M., An O. (2019). Identification of a novel enhancer of CEBPE essential for granulocytic differentiation. Blood.

[39] Nakajima H., Watanabe N., Shibata F., Kitamura T., Ikeda Y., Handa M. (2006). N-terminal Region of CCAAT/Enhancer-binding Protein epsilon is critical for cell cycle arrest, apoptosis, and functional maturation during myeloid differentiation. J. Biol. Chem..

[40] Wells J., Boyd K.E., Fry C.J., Bartley S.M., Farnham P.J. (2000). Target gene specificity of E2F and pocket protein family members in living cells. Mol. Cell. Biol..

[41] Takahashi Y., Rayman J.B., Dynlacht B.D. (2000). Analysis of promoter binding by the E2F and pRB families in vivo: Distinct E2F proteins mediate activation and repression. Genes Dev..

[42] Becker-Hapak M., McAllister S.S., Dowdy S.F. (2001). TAT-mediated protein transduction into mammalian cells. Methods.

