# Supplementary material for: CCAAT/Enhancer-Binding Protein ε27 Antagonism of GATA-1 Transcriptional Activity in the Eosinophil Is Mediated by a Unique N-Terminal Repression Domain, Is Independent of Sumoylation and Does Not Require DNA Binding
Source: Int J Mol Sci. 2021 Nov 24;22(23):12689. doi: 10.3390/ijms222312689 (PMC8657826; doi:10.3390/ijms222312689)
Supplement: Supplementary file 1 [file ijms-22-12689-s001.zip › ijms-1441101-supplementary.pdf]

Supplemental Figures:

A

Domain/Function	C/EBPε Isoform 32/30kD	27kD	14kD
DNA Binding	+	+	+
Transactivation	+	+	-
Dimerization	+	+	+
Function	Activation	Repression?	Repression?

B

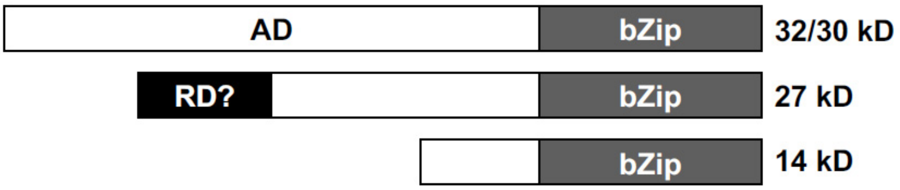

Figure S1. Structure, functional domains and likely activities of the C/EBPε isoforms. (A) The C/EBPε 32/30kD, 27kD and 14kD isoforms are generated by alternative promoter usage, mRNA splicing, and translational start sites. AD = activation domain; RD = hypothesized repressor domain, a region distinct from the 32 and 30 kD isoforms. (B) The presence of DNA-binding, transactivation and b-Zip dimerization domains, and known and hypothetical function is summarized for the various C/EBPε isoforms.

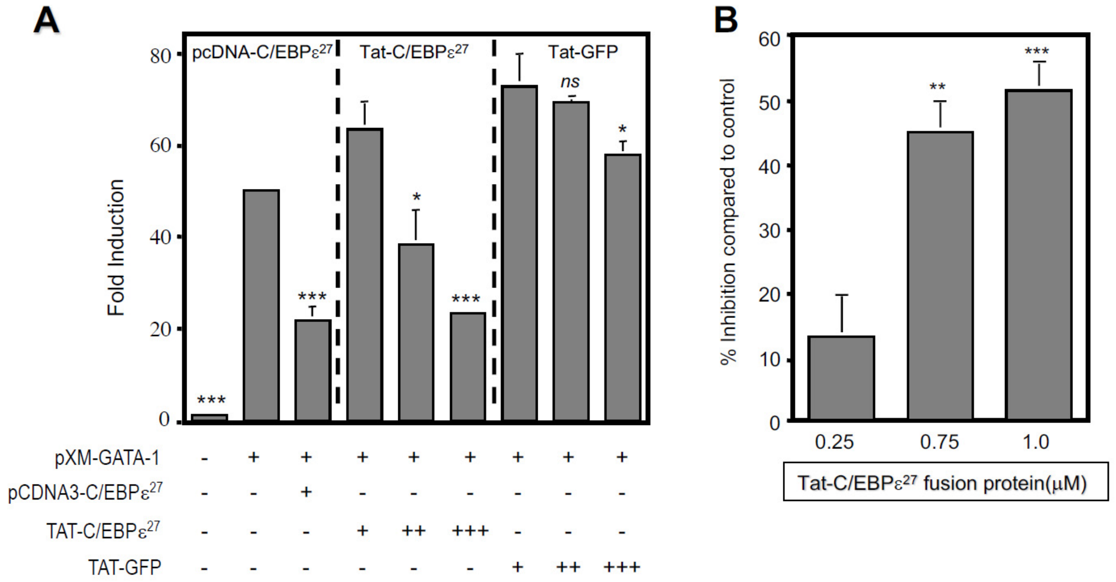

Figure S2. TAT-C/EBPε[27] inhibits GATA-1 transactivation of the MBP1-P2 promoter in CV-1 cells. (A) CV-1 cells were transduced for 30 min. with TAT-C/EBPε[27] or the TAT-GFP control fusion protein, followed by Fugene™-mediated transfection with GATA-1 expression vector (pXM-GATA-1) and the pXP2-MBP1-P2 luciferase reporter plasmid. For comparison, non-transduced cells were transfected at the same time with the expression vectors for C/EBPε[27] (pcDNA3-C/EBPε[27]), GATA-1 (pXM-GATA-1) and the pXP2-MBP1-P2 promoter luciferase reporter plasmid. Luciferase activities analyzed at 24 hrs were normalized to and are displayed as fold induction over the pXP2-MBP1-P2 luciferase reporter alone. (****p*,0.001; **p*<0.05; *ns*, not significant, compared to GATA-1 alone). (B) Dose-dependent inhibition of GATA-1 transactivation of the MBP1-P2 promoter in CV-1 cells transduced with 0.25 - 1.0μM TAT-C/EBPε[27]. CV-1 cells were transduced with TAT-C/EBPε[27] or TAT-GFP control protein for 30 min. followed by Fugene™-mediated transfection with pXM-GATA-1 and the MBP1-P2 reporter plasmid. Results were normalized to MBP1-P2 promoter activity in cells transduced with the TAT-GFP control protein, and are plotted as % inhibition compared to the control. Representative results for 3 independent experiments are shown (****p*<0.001; ***p*<0.01, compared to 0.25μM dose).

.
